# The Importance of the CXCL12/CXCR4 Axis in Therapeutic Approaches to Diabetes Mellitus Attenuation

**DOI:** 10.3389/fimmu.2015.00403

**Published:** 2015-08-07

**Authors:** Melita Vidaković, Nevena Grdović, Svetlana Dinić, Mirjana Mihailović, Aleksandra Uskoković, Jelena Arambašić Jovanović

**Affiliations:** ^1^Department of Molecular Biology, Institute for Biological Research, University of Belgrade, Belgrade, Serbia

**Keywords:** CXCL12, CXCR4, CXCR7, diabetes mellitus, diabetic complications, pancreatic β-cells

## Abstract

The pleiotropic chemokine (C–X–C motif) ligand 12 (CXCL12) has emerged as a crucial player in several diseases. The role of CXCL12 in diabetes promotion and progression remains elusive due to its multiple functions and the overwhelming complexity of diabetes. Diabetes is a metabolic disorder resulting from a failure in glucose regulation due to β-cell loss and/or dysfunction. In view of its ability to stimulate the regeneration, proliferation, and survival of β-cells, as well as its capacity to sustain local immune-isolation, CXCL12 has been considered in approaches aimed at attenuating type 1 diabetes. However, a note of caution emerges from examinations of the involvement of CXCL12 in the development of diabetes and its complications, as research data indicate that CXCL12 displays effects that range from protective to detrimental. Therefore, as a beneficial effect of CXCL12 in one process could have deleterious consequences in another, a more complete understanding of CXCL12 effects, in particular its functioning in the cellular microenvironment, is essential before CXCL12 can be considered in therapies for diabetes treatment.

## Introduction

The worldwide prevalence of diabetes has increased six-fold over the past 20 years, with diabetes assuming the proportions of an epidemic. Therefore, research devoted toward improving the steps that could be undertaken in the prevention and treatment of diabetes and its complications is a scientifically and socially significant task. Diabetes mellitus is a complex metabolic disorder that is presented in two major forms, type 1 diabetes (T1D) and the more common type 2 diabetes (T2D). While these conditions have different etiologies, both types of diabetes are characterized by hyperglycemia resulting either from insufficient insulin levels as in T1D, or by an insensitivity of target cells to insulin as in T2D. In T1D, hyperglycemia occurs as a result of destruction of insulin-producing pancreatic β-cells in an autoimmune process. T2D is a metabolic disease characterized by β-cell dysfunction and peripheral insulin resistance. Genetic predisposition and environmental factors, such as diet, physical inactivity, and viral infections contribute to the etiology of diabetes. Early exposure to hyperglycemia predisposes individuals to the development of diabetic complications, a phenomenon which is referred to as “metabolic memory” (1). At present, diabetes management is focused on lowering hyperglycemia and treating its pathological consequences, rather than its initial triggers.

## CXCL12 and its Receptors

Current research has positioned CXCL12 as an important molecule in potential treatment of diabetes and its complications. CXCL12 belongs to the CXC group of chemokines. It is a potent chemoattractant involved in angiogenesis, leukocyte trafficking, cancer, inflammatory disorders, atherosclerosis, and HIV pathology (2, 3). CXCL12 is a ligand for two transmembrane receptors: CXCR4 and CXCR7 (4, 5). Interaction between CXCL12 and its receptor CXCR4 induces downstream signaling involved in chemotaxis, cell survival, proliferation, increase in intracellular calcium, and gene transcription (3). CXCR7 is an atypical chemokine receptor that does not signal through the canonical G-protein pathway. CXCL12 binds CXCR7 with even higher affinity than CXCR4. CXCR7/CXCL12 interaction triggers CXCR7 association with β-arrestin 2 and CXCL12/CXCR7 internalization, implying capability of CXCR7 to decrease the level of CXCL12 from the surroundings (6). The observed promotion of cell survival, adhesion, and tumor growth by CXCR7 points to signaling pathways that lie downstream of this receptor (7). This may be in correlation with its ability to heterodimerize with CXCR4 and regulate CXCR4/CXCL12-mediated processes. The role of CXCR7 in mediating the anti-apoptotic effect of CXCL12 has been documented (8). CXCR7 has also emerged as a determinant of autoimmunity and β-cell destruction which underlies diabetic progression (9).

## The Role of CXCL12/CXCR4 Axis in Diabetes Pathophysiology

### Type 1 diabetes

Type 1 diabetes is an autoimmune disease triggered by environmental factors in genetically susceptible persons. In T1D, pancreatic β-cells are targeted by the individual’s own immune system resulting in reduced or complete elimination of insulin production. Discouraging, long-term studies of islet transplantation stress the need for new strategies to counteract autoimmunity, and CXCL12 has emerged as a key molecule in this process. CXCL12 plays a particularly important role in directing T cell migration and therefore in immune processes. Recruitment of autoreactive T cells into pancreatic islets leads to inflammation (referred to as insulitis) that initiates T1D development. Several reports have revealed that neutralization of CXCL12 inhibits insulitis and diabetes development (10, 11). It was proposed that retention of regulatory T cells (Tregs) in the bone marrow by CXCL12 disturbs the balance of T cells in favor of autoreactive T cells, which intensifies disease progress. However, a reverse effect of CXCL12 inhibition was reported by Aboumarad et al. who showed that a population of CXCR4+ T cells attracted by CXCL12 protects recipient mice from the adoptive cell transfer of diabetes (12). The beneficial effects of CXCL12 could be explained by several properties specific to this chemokine. CXCL12 induces bi-directional movement of T cells, toward lower concentration and away from higher CXCL12 concentration (13). CXCL12 also exerts a chemorepulsive effect on diabetogenic T cells, while mediating firm adhesion of normal T cells (14). Moreover, CXCL12 expression in islets was shown to cause the selective repulsion of autoreactive T cells and retention of Tregs at the site (15). Tregs play a crucial role in suppressing autoimmunity and data support their relevance in T1D pathogenesis (16). It was reported that pancreatic lymph nodes (PLNs) of non-obese diabetic (NOD) mice lack Tregs, while the recovery of euglycemia in these mice was associated with the restoration of the Treg population in PLNs (17). The absence of Tregs correlates with the locally decreased expression of CXCL12, suggesting that improved function of the CXCL12/CXCR4 axis and subsequent retention of Tregs in the PLNs could serve as the basis for an alternative therapeutic approach for treating T1D. Selective repulsion of autoreactive T cells and attraction of Tregs have been proposed as a mechanism for a recently reported novel strategy in islet transplantation. It has been shown that immune-mediated rejection of transplanted islets could be delayed by their local immune-isolation achieved through coating or encapsulation of islets with CXCL12, thus excluding the need for systemic immunosuppression (18). Despite the evidence importance of this finding for T1D treatment, it should be noted that local immunosuppression achieved through CXCL12 has also been observed in cancer models where this mechanism protects cancer cells from immune attack (19).

### Type 2 diabetes

Type 2 diabetes is a metabolic disorder characterized by insulin resistance in adipose tissue, liver and skeletal muscle, and defective pancreatic insulin secretion. Experimental and clinical data describe diabetes as a chronic inflammatory disease (20). More than 20 years ago, it was shown that the pro-inflammatory cytokine TNF-α was capable of inducing insulin resistance (21). It is now known that the plasma levels of pro-inflammatory cytokines, such as TNF-α, IL-6 or IL-1β, and chemokines are elevated in T2D patients, while *in vivo* studies have revealed that inhibition of key inflammatory cytokines protects rodents from insulin resistance (22). The inflammatory cytokines promote insulin resistance by interfering with insulin signaling through activation of JNK kinase and NFκB pathways (23). Pancreatic β-cells respond to insulin resistance by increasing insulin secretion. However, when β-cells fail to compensate for increased insulin demands, T2D develops.

Chronic low levels of inflammation in the pancreas and insulin-responsive tissues of diabetics are accompanied by infiltration of lymphocytes and macrophages. The latter process is associated with a switch from an anti- to a pro-inflammatory profile. Namely, diabetes is linked to a disturbed balance between pro-inflammatory (Th1 and Th17) and anti-inflammatory (Th2 and Tregs) subsets of T cells in favor of the pro-inflammatory phenotype. As a result, Th1 and Th17 promote the polarization of M1 macrophages, which are the main producers of pro-inflammatory cytokines (24). CXCL12 has a controversial role in inflammation, as a result of its ability to orchestrate the trafficking of a variety of immune cells. Based on the reports describing CXCL12-promoted recruitment of immune cells to inflamed tissues in autoimmune diseases such as rheumatoid arthritis (RA) and lupus erythematosus in lung inflammation and inflammatory bowel disease (25), CXCL12 has been proposed to have a pro-inflammatory role. Also, CXCL12 recruits monocytes into adipose tissue, which after differentiation secrete pro-inflammatory cytokines in obesity. It was suggested that the CXCL12/CXCR4 axis induces M1 macrophage accumulation, subsequent inflammatory cytokine production, and finally insulin resistance (26). However, CXCL12 was also found to possess an anti-inflammatory role by mediating T cell polarization toward Tregs, and by stimulating IL-10 production in anti-inflammatory M2 macrophages (27). Moreover, CXCL12 promotes migration of monocytes and their polarization toward the M2 phenotype (28), which points to the potential role of CXCL12 in reducing inflammation in diabetes. Once again, we should be aware that promotion of the anti-inflammatory Treg/M2 phenotype by CXCL12 is part of the mechanism involved in suppression of anti-tumor immunity mediated by this chemokine (29).

Given the strong correlation between inflammation and T2D, anti-inflammatory strategies for T2D treatment have been proposed and some have been clinically tested. Encouraging results were observed with salsalate, an inhibitor of the NF-κB pathway, and IL-1β antagonists. Potential targeting of CXCL12 for T2D treatment requires additional studies and a better understanding of the role of CXCL12 in T2D and inflammation.

## Potential Utilization of the CXCL12/CXCR4 Axis in Diabetes Management through Promotion of Pancreatic β-Cell Differentiation, Regeneration, and Survival

Current limitations in diabetes treatment have stimulated efforts toward β-cell replacement therapy. Preservation of β-cell mass, stimulation of β-cell differentiation from embryonic stem cells (ESCs), regeneration of the impaired endocrine pancreas from remaining β-cells, and cellular reprograming of other endocrine or exocrine cell types in pancreas could provide a long-term solution in diabetes treatment (30–32). This strategy requires understanding of the molecular mechanisms that control β-cell maturation, growth, and survival.

### The CXCL12/CXCR4 axis in β-cell differentiation and regeneration

CXCL12/CXCR4 signaling is crucial for β-cell differentiation and pancreatic islet genesis (31). CXCL12 is expressed in the gut endoderm and attracts CXCR4 expressing angioblasts which induce pancreatic and duodenal homeobox 1 (Pdx1) expression in the pre-pancreas region (33). Pdx1 plays a key role in the expression of neurogenin 3 (Ngn3) which is essential for the formation of all islet cell types (34). During human fetal β-cell development, CXCR4 is necessary for the *in vivo* differentiation of islet-like clusters into β-cells while CXCL12 directs the proliferation of epithelial endocrine precursors through activation of phosphatidyl inositol (PI)-3 and Akt kinases (31). Expression of interferon (IFN) γ, which is under the control of the insulin promoter, promotes ductal hyperplasia and regeneration of new islets in the pancreas of transgenic mice (35), providing an excellent model for studding pancreas regeneration. When NOD mice were used as an IFNγ transgenic model, their pancreas displayed three- and four-fold elevated expression of CXCL12 and CXCR4, respectively, in comparison to non-transgenic NOD mice (36). CXCL12 expression in IFNγ-NOD mice stimulated pancreatic ductal cell migration and activation of the Akt, Src, and extracellular signal regulated protein kinase (ERK1/2) in duct cells, revealing the essential role of CXCL12 in their survival, proliferation, and migration during pancreatic regeneration. These insights could help in developing new therapeutic protocols for stimulating the differentiation of pancreatic stem cells into β-cells, proliferation of existing β-cells and transdifferentiation of particular cell types in diabetic patients (31, 32, 37). Furthermore, *in vitro* treatment of human ESCs with appropriate signals could direct their differentiation into β-cells that could be transplanted in diabetic patients.

### The CXCL12/CXCR4 axis in β-cell survival

Diabetes-related studies have provided evidence for an important role of CXCL12 in anti-apoptotic and anti-necrotic protection of β-cells from diabetogenic agents. Transgenic mice overexpressing CXCL12 in β-cells are protected from streptozotocin (STZ)-induced diabetes via activation of Akt kinase (38). CXCL12-stimulated Akt signaling activates anti-apoptotic signals in β-cells through increased expression of the anti-apoptotic B cell lymphoma 2 (Bcl-2) protein and anti-apoptotic phosphorylation of the proapoptotic Bcl-2-associated death (BAD) protein. CXCL12-mediated induction of Akt activity also promotes activation and stabilization of beta-catenin/transcription factor 7-like 2 (TCF7L2) that contributes to the survival of isolated islets and INS-1 cells (39). Activation of the CXCL12/CXCR4 axis by STZ, cytokines and in thapsigargin injury of human and mouse islets promotes intra-islet GLP-1 production and enhances β-cell survival (40). It has been proposed that the paracrine action of CXCL12 from β-cells activates Akt in adjacent α-cells, promoting their proliferation and production of GLP-1 instead of glucagon. CXCL12 and GLP-1 signal from α-cells together control the growth and viability of β-cells. These findings raise the possibility that the anti-apoptotic/prosurvival CXCL12 and progrowth GLP-1 signaling act either additively or synergistically to conserve or possibly enhance β-cell mass in response to injury.

**Figure 1:**
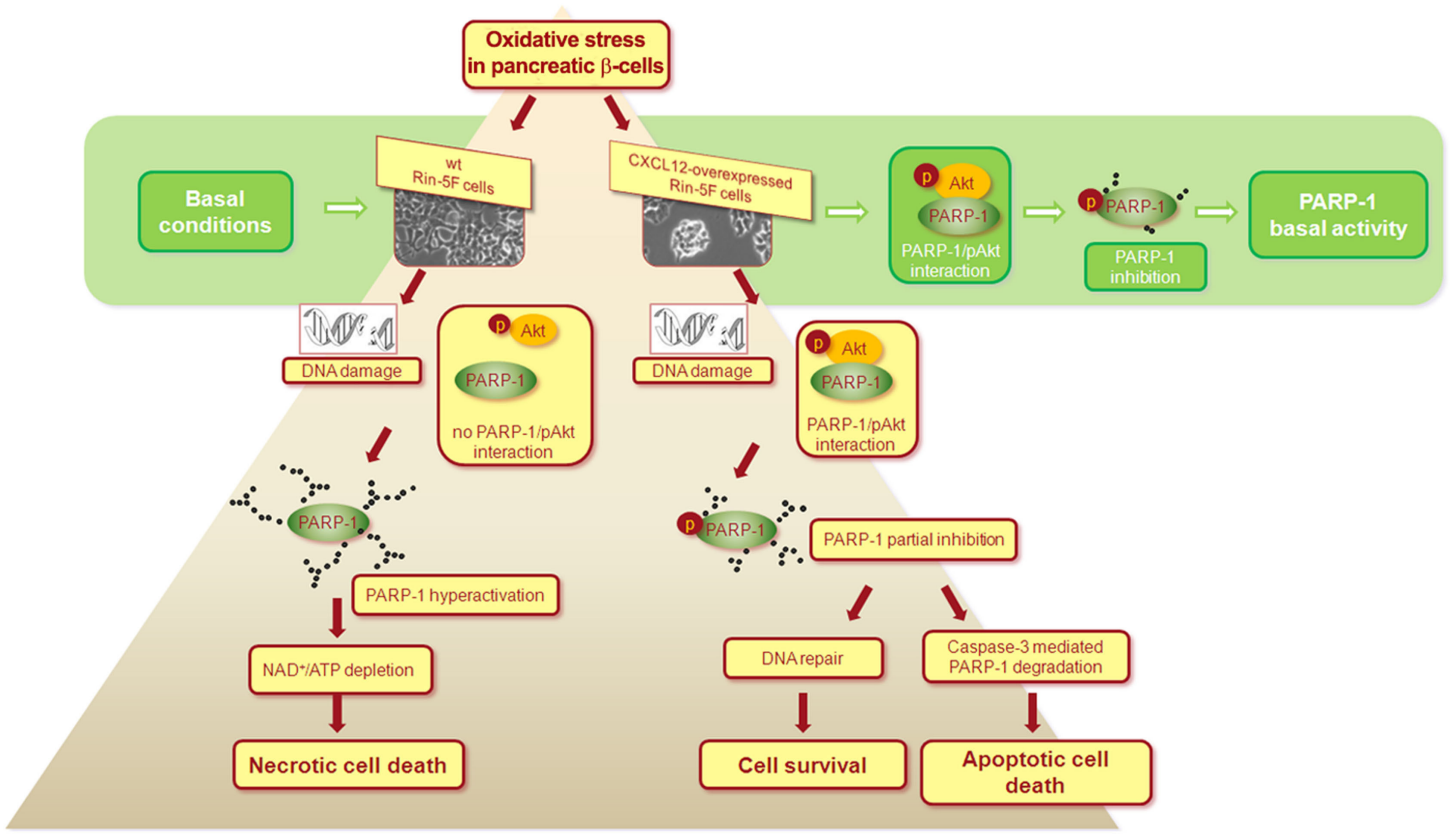
**Proposed mechanism of the CXCL12/Akt-mediated anti-necrotic effect that leads to pancreatic **β**-cells survival**. Under basal conditions, interaction between activated Akt (pAkt) and PARP-1 leads to PARP-1 phosphorylation that results in partial inhibition of PARP-1 in wild type (wt) and CXCL12 overexpressing Rin-5F cells. After hydrogen peroxide-induced oxidative stress and in response to severe DNA damage in wt cells, the loss of pAkt-PARP-1 interaction allows PARP-1 hyperactivation, followed by extensive PARP-1 auto-poly(ADP-ribosyl)ation, NAD^+^ and ATP depletion, and final necrotic cell death. In CXCL12 overexpressing cells, pAkt-PARP-1 interaction persists after hydrogen peroxide treatment, maintaining partial inhibition of PARP-1. Consequently, cellular energy depletion is prevented and a switch from the necrotic to the apoptotic cell death is ensured. With CXCL12-mediated suppression of PARP-1 overactivation in stress conditions, cell still operates with the active PARP-1 that is essential player in many cellular processes. The mechanism is based on the findings presented in Ref. (41).

CXCL12 overexpression considerably improves insulin expression and viability of isolated rat islet cells and Rin-5F pancreatic β-cells after hydrogen peroxide treatment (41). CXCL12 overexpression in pancreatic cells switches hydrogen peroxide-induced cell death from the necrotic to the apoptotic pathway through Akt kinase-mediated reduction of poly(ADP-ribose) polymerase-1 (PARP-1) activity (Figure 1). These findings are in correlation with the documented role of PARP-1 in necrotic cell death (42) and with the observation that pharmacological inhibition or genetic deletion of PARP-1 protects animals against chemically induced and spontaneous diabetes development (43). The anti-necrotic effect of CXCL12 could prevent an additional pro-inflammatory burden of β-cells provoked by necrosis and could therefore be used for diabetes treatment.

### Transcriptional regulation of the CXCL12 gene

Considering the involvement of CXCL12 in β-cell differentiation, growth, and survival, an understanding of CXCL12 transcriptional regulation offers possibility to improve β-cell mass in diabetes. PARP-1 is involved in rat CXCL12 gene (*Cxcl12*) downregulation during the early stage of STZ-induced oxidative stress (44). During later stages of oxidative stress and intensive pancreatic β-cell injury, *Cxcl12* expression is upregulated by Yin Yang 1 (YY1). Multiple protein–protein interactions between C/EBPα, C/EBPβ, STAT3, p53, FOXO3a, and HMG I/Y transcription factors that bind to the *Cxcl12* promoter in the rat pancreatic Rin-5F cell line suggest that multi-subunit protein complexes are responsible for the regulation of *Cxcl12* transcription (44). Targeted stimulation or suppression of specific transcription factors involved in the regulation of genes engaged in the proper functioning of β-cells could improve therapeutic approaches in diabetes.

## The Controversial Role of CXCL12 in Diabetic Complications

Along with the promotion of cell survival, the biological effects of CXCL12, such as angiogenesis induction and recruitment of bone marrow-derived progenitor cells suggest that this chemokine assumes a central position in tissue repair and regeneration. Since diabetes is accompanied by dysfunction and life-threatening damage of several organs, the potential of CXCL12 to create a microenvironment that supports repair processes is particularly important. Indeed, CXCL12 accelerates wound healing in diabetes by recruiting endothelial progenitor cells (EPCs) and through improved angiogenesis (45, 46). However, the same injury response mechanisms could also promote disease progression, as in diabetic retinopathy, which starts with damage to small blood vessels in the eye and leads to reduced blood flow and ischemia. The ischemia promotes aberrant neovascularization that destroys the normal retinal architecture, causing impaired vision. In agreement with the fact that the expression of CXCL12 is controlled by hypoxia-inducible factor-1 (47), the level of CXCL12 increases as diabetic retinopathy progresses and contributes to angiogenesis by recruiting EPCs to the site of vascular injury (48). Blocking the function of CXCL12 prevents neovascularization and progression of proliferative retinopathy.

Diabetic nephropathy is characterized by the development of albuminuria with a subsequent decline in glomerular filtration rate, usually followed by failure of renal function. CXCL12 expression in the kidney increases during acute renal failure, resulting in homing of progenitor cells to the injured kidney (49). Although CXCL12 is considered as one of the major mediators involved in kidney repair after ischemic acute renal failure, data regarding the role of this chemokine in diabetic nephropathy are limited. A study on the mouse model of T2D revealed that CXCL12 contributes to glomerulosclerosis, podocyte loss, and albuminuria, implicating the pathogenic role of CXCL12 in diabetic nephropathy (50). The same group proposed a novel strategy for the prevention of glomerulosclerosis in T2D. This is based on the protective effect of dual chemokine CCL2-CXCL12 blockage: inhibition of CCL2-mediated glomerular leukocyte recruitment and CXCL12-mediated loss of podocytes (51).

Diabetes dramatically increases the risk of various cardiovascular problems, including coronary artery disease with myocardial infarction and atherosclerosis (52). Although the importance of CXCL12 in cardiovascular disease has been intensively studied, current findings once again suggest a double-edged role of this chemokine in ischemic heart and atherosclerosis (53). Since this issue has been extensively reviewed elsewhere in Ref. (53), it will not be considered here in more detail. It is worth mentioning that genome-wide association studies revealed a significant association of two SNPs downstream of the *CXCL12* gene with cardiovascular disease (54).

The general conclusion regarding the role of CXCL12 in diabetic complications is that CXCL12 walks a thin line between protective and detrimental effects. Therapies that rely on either promotion of CXCL12 or blocking of its activity have been suggested for a variety of conditions. The above-mentioned findings indicate that CXCL12-based therapy should be used with extreme caution and by precise targeting of CXCL12 action to specific tissue (Table 1). Proper functioning of the immune system provides a balance between protection from pathogens and tissue damage, and between autoimmunity and cancer suppression. Disturbance of this balance might be beneficial in one process, while at the same time detrimental in another.

**Table 1:** **Yin-Yang nature of CXCL12 in diabetic complications**.

Effects of activated CXCL12/CXCR4 axis (in a mouse model system)	Role of the CXCL12/CXCR4 axis in diabetes	Reference
Accelerates wound healing in diabetes, improves angiogenesis	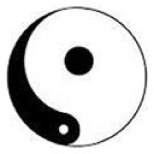	(45, 46)
Promotes diabetic retinopathy, contributes to angiogenesis via recruitment of EPCs to the site of vascular injury	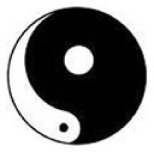	(48)
Improves diabetes progression in NOD mice by sequestering Tregs in the bone marrow, which disturbs the balance in favor of autoreactive T cells	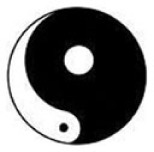	(10)
Prevents insulitis and autoimmune diabetes via recruitment of Th2-type cells to the pancreas of NOD mice	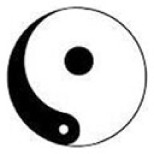	(12)
Mediates kidney repair by homing of progenitor cells to the injured kidney in acute renal failure	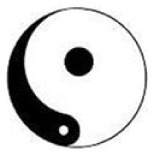	(49)
Contributes to progression of diabetic nephropathy through involvement in glomerulosclerosis, podocyte loss and albuminuria	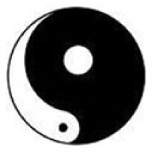	(50)
Induces M1 macrophage accumulation in adipose tissue which leads to secretion of pro-inflammatory cytokines in obesity, associated with insulin resistance	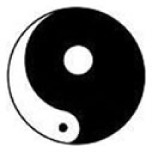	(26)

*Balanced (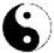) CXCL12 expression and distribution is indispensable for the final outcome of physiological action that can produce either protective (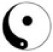) or detrimental effects (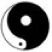) during diabetes onset and in diabetic complications*.

## Conclusion

The role of CXCL12 in diabetes is very complex. For a better understanding of the biological effects of CXCL12, additional studies are needed to clarify several issues. As has already been mentioned, aside from CXCR4, CXCL12 also binds to its second receptor CXCR7, whose downstream signaling is elusive. The reported heterodimerization of these receptors has introduced additional complexity to CXCL12 signaling (55). CXCR7 also binds CXCL11 (56), while CXCR4 has been shown to be a receptor for the cytokine MIF and chemokine CXCL14 (57, 58). Therefore, the biological functions of CXCL12 must be considered in the context of a specific microenvironment, taking into account the site of CXCL12 expression, the expression of other chemokines, and all receptors on target cells. A complete understanding of the complex CXCL12 network is a prerequisite for the safe application of CXCL12-based therapy in diabetes.

## Conflict of Interest Statement

The authors declare that the research was conducted in the absence of any commercial or financial relationships that could be construed as a potential conflict of interest.
